# Transcriptome analysis of messenger RNA and long noncoding RNA related to different developmental stages of tail adipose tissues of sunite sheep

**DOI:** 10.1002/fsn3.2537

**Published:** 2021-08-25

**Authors:** Xige He, Rihan Wu, Yueying Yun, Xia Qin, Lu Chen, Yunfei Han, Jindi Wu, Lina Sha, Gerelt Borjigin

**Affiliations:** ^1^ College of Food Science and Engineering Inner Mongolia Agricultural University Hohhot China; ^2^ College of Biochemistry and Engineering Hohhot Vocational College Hohhot China; ^3^ School of Life Science and Technology Inner Mongolia University of Science and Technology Baotou China

**Keywords:** long noncoding RNA, Sunite sheep, tail fat, transcriptome analysis

## Abstract

The tail fat of sheep is the most typical deposited fat, and it can be widely used in human daily life, such as diet, cosmetics, and industrial raw materials. To understand the potential regulatory mechanism of different growth stages of tail fat in Sunite sheep, we performed high‐throughput RNA sequencing to characterize the long noncoding RNA (lncRNA) and messenger RNA (mRNA) expression profiles of the sheep tail fat at the age of 6, 18, and 30 months. A total of 223 differentially expressed genes (DEGs) and 148 differentially expressed lncRNAs were found in the tail fat of 6‐, 18‐, and 30‐month‐old sheep. Based on functional analysis, we found that fat‐related DEGs were mainly expressed at 6 months of age and gradually decreased at 18 and 30 months of age. The target gene prediction analysis shows that most of the lncRNAs target more than 20 mRNAs as their transregulators. Further, we obtained several fat‐related differentially expressed target genes; these target genes interact with different differentially expressed lncRNAs at various ages and play an important role in the development of tail fat. Based on the DEGs and differentially expressed lncRNAs, we established three co‐expression networks for each comparison group. Finally, we concluded that the development of the sheep tail fat is more active during the early stage of growth and gradually decreases with the increase in age. The mutual regulation of lncRNAs and mRNAs may play a key role in this complex biological process.

## Graphical abstract

The mRNA and lncRNA expression profiles were characterized in three different growth stages of Sunite sheep fat tail tissue. The development of sheep tail fat is more active in the early stage and gradually decreases with the increase of age. LncRNA may regulate the development of Sunite sheep tail fat mainly through positive correlation with multiple mRNAs.

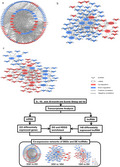

## 1 INTRODUCTION

Adipose tissue is found in various parts of the sheep body, some of which are the subcutaneous layer under the skin, around the kidneys, within the abdominal cavity, and tail. The tail fat is the most typical deposited fat, especially in the several Mongolian sheep breed, such as Sunite Sheep (SS), Wuzhumuqin Sheep, and Wuranke Sheep, which are several fat‐tailed sheep species in China. Among them, SS is the representative of local elite breeds in Inner Mongolia, and it is the primary meat resource with the benefits of great numbers, delicate flesh, and good flavor, which is very popular among consumers. SS is mainly raised in the Xilingol grassland of Inner Mongolia. These sheep are accustomed to voluntary movement and typically free‐feed (or naturally graze) throughout their lives. SS is a meat breed, and the important phenotype of SS is the fat tail. With the growth of SS, the tail fat increases continuously and reaches the weight of about 3–4.5 kg at 30 months of age (30 M). Tail fat can be used by humans as an important source of dietary fat (Kashan et al., 2005; Moradi et al., 2012) and provides the energy needed by the human body. As a by‐product of mutton, it can also be used as a raw material for daily‐use products, such as soap, cosmetics, and medicinal materials.

Adipose tissue plays a vital role in maintaining the balance of homeostatic metabolic processes in domestic animals. During severe conditions, such as food scarcity resulting from migration, drought, and winter, the tail fat can provide energy (Ben Sassi‐Zaidy et al., 2014). According to a previous study (Kashan et al., 2005), fat‐tailed sheep have a low percentage of intramuscular fat and provide good quality lean meat. In contrast, short‐tailed sheep have higher intramuscular fat storage. Thus, the mechanism of tail fat deposition is worth studying. Many studies have employed RNA sequencing (RNA‐seq) to explore differentially expressed genes (DEGs) in the adipose tissues in fat‐tailed sheep recently. To gain a better understanding of fat deposition, Li et al. (2018a, 2018b) performed RNA‐seq of perirenal, subcutaneous, and tail fat tissues from Guangling Large‐Tailed and Small‐Tailed Han (STH) sheep to determine their transcriptome profiles. The result showed that a total of 4131 DEGs were identified in tail fat tissue, and 49 genes were shown to be involved in the peroxisome proliferator‐activated receptor (PPAR) signaling pathway, which is the key pathway to balance fat metabolism (Corrales et al., 2018). Wang et al., (2014) used transcriptome sequencing to compare the transcriptome profiles of tail fat tissue between Kazak and Tibetan sheep. This study identified 646 DEGs between the two breeds, and the top two genes with the largest fold change (*NELL1* and *FMO3*), which may be relevant to fat metabolism in adipose tissues. Further, the lncRNAs and mRNAs associated with tail fat deposition and development in Lanzhou fat‐tailed sheep (long fat‐tailed sheep), STH sheep (thin‐tailed sheep), and Tibetan sheep (short thin‐tailed sheep) were analyzed; 407 DEGs and 68 differentially expressed (DE) lncRNAs were identified (Ma et al., 2018). It was shown that the DEGs and target genes of DE lncRNAs were enriched in fatty acid metabolism and fatty acid elongation‐related pathways through gene ontology (GO) analysis and Kyoto encyclopedia of genes and genomes (KEGG) pathway analysis, which contribute to fat deposition. Network contribution based on DE mRNA and lncRNAs shows that some DE lncRNAs (*TCONS_00372767*, *TCONS_00171926*, *TCONS_00054953*, and *TCONS_00373007*) may play an important role in tail fat deposition processes. Bakhtiarizadeh and Salami (2019) have performed the transcriptome analysis in fat‐tailed (Lori‐Bakhtiari) and thin‐tailed (Zel) Iranian sheep breeds and identified 7 DE lncRNAs and 311 DEGs between the two breeds. Further, the target prediction analysis shows that the novel lncRNAs can regulate the expression of genes involved in lipid metabolism through cis‐ or transregulation. In addition, the animal quantitative trait loci database suggested 1 intronic and 6 intergenic lncRNAs as candidates of sheep fat‐tail development. Transcriptome analyses were performed in specific sheep tissues to reveal the potential regulatory roles of lncRNAs, such as in the skeletal muscle (Chao et al., 2018; Li et al. 2018a, 2018b; Wei et al., 2018), pituitary (Li et al., 2020; Yang et al., 2020; Zheng et al., 2019), testis (Yang et al., 2018; Zhang et al., 2017), and ovaries (Feng et al., 2018; Miao, Luo, Zhao, & Qin, 2016a, 2016b). However, related research on mRNA and lncRNA in SS tail fat is still lacking, including the regulation mechanism of fat deposition and related molecular pathways of tail fat development. To better understand the potential role of mRNAs and lncRNAs in fat‐tailed sheep, we explored the transcriptomic differences in SS’s tail fat at three different growth stages, 6 months of age (6 M), 18 months of age (18 M), and 30 M. This facilitated the characterization of the mRNA and lncRNA expression profiles in the fat tail of SS and elucidate the molecular mechanism of fat deposition. Our findings may lay a foundation for further studies in fat‐tailed sheep. In particular, our study provides some information on the mechanism of fat development in fat‐tailed sheep during different growth processes, which is of great significance for the development and utilization of by‐products of meat breeds of sheep.

## 2 MATERIALS AND METHODS

### 2.1 Animal and tail fat tissue collection

Nine castrated Sunite rams were selected from three different growth stages, 6 M (*n* = 3), 18 M (*n* = 3), and 30 M (*n* = 3), respectively. All sheep were raised under the same conditions, including food, water source, and environment. After slaughtering, adipose tissue was sampled from the tail fat (top 1/3) and cut into small pieces of 2 mm × 2 mm × 2 mm (Miao et al., 2015). These small pieces were immediately placed into cryotube (sterile without enzyme), frozen in liquid nitrogen, and transferred to −80°C until RNA extraction.

### 2.2 RNA extraction and RNA‐seq

Total RNA from the nine adipose tissue samples was extracted using TRIzol reagent (Invitrogen, CA, USA) according to the manufacturer's procedure. Quantity and purity of the total RNA were analyzed with Bioanalyzer 2100 and RNA 6000 Nano LabChip Kit (Agilent, CA, USA), respectively, with RNA integrity number >7.0. Approximately 10 µg of total RNA was used to deplete ribosomal RNA by following the manufacturer's instructions of the Epicentre Ribo‐Zero Gold Kit (Illumina, San Diego, USA). After purification, divalent cations were applied to fragment poly (A) tailor poly (A)+ RNA fractions into small pieces under high temperature. Then, the cleaved RNA fragments were reverse transcribed to create the final complementary DNA (cDNA) library according to the protocol for the mRNA‐Seq sample preparation kit (Illumina, San Diego, USA), and the average insert size for the paired‐end libraries was 300 bp (±50 bp). Eventually, the paired‐end sequencing was performed following the vendor's recommended protocol of the Illumina Hiseq 4000.

### 2.3 Transcripts assembly

The FastQC (http://www.bioinformatics.babraham.ac.uk/projects/fastqc/) software was used to verify the sequence quality, and adaptor contamination, low‐quality bases, and undetermined bases in the raw data were removed by the Cutadapt software (Martin, 2011). The clean reads were mapped into the genome of sheep (*Ovis aries v3.1)* using Bowtie2 (Langmead & Salzberg, 2012) and Tophat2 (Kim et al., 2013), and mapped reads were assembled using the StringTie software (Pertea et al., 2015). To reconstruct a comprehensive transcriptome, all transcriptomes from sheep samples were merged using Perl scripts. After the generation of the final transcriptome, the expression levels of all transcripts were estimated using the StringTie and R package Ballgown (Frazee et al., 2015).

### 2.4 lncRNA identification and different expression analysis

First, transcripts that overlapped with known mRNAs and transcripts smaller than 200 bp were excluded. Subsequently, the Coding Potential Calculator (CPC) (Kong et al., 2007) and Coding‐Non‐Coding Index (CNCI) software tools (Sun et al., 2013) along with Pfam database (Punta et al., 2012) were utilized to predict transcripts with coding potential. Transcripts that scored CPC < −1 and CNCI < 0 were discarded. The remaining transcripts with class code (i, j, o, u, x, =) were considered as lncRNAs. The definition of class code is as follows: (i) a transcript falling entirely within a reference intron (intronic); (j) potentially novel isoform or fragment at least one splice junction is shared with a reference transcript; (o) generic exonic overlap with a reference transcript; (u) unknown, intergenic transcript (intergenic); (x) exonic overlap with reference on the opposite strand (antisense); (=) complete match, considered as known lncRNA. Expression levels of lncRNAs and mRNAs were calculated as fragments per kilobase of transcript per million mapped reads using the StringTie. The DE mRNAs and lncRNAs were determined with an absolute value of log_2_(Fold Change) ≥1 and false detection rate (FDR) <0.05 using the Ballgown (Frazee et al., 2015).

### 2.5 Target gene prediction and functional analysis of lncRNAs

In order to explore the functions of lncRNAs, the DE lncRNAs were analyzed for target prediction. In this study, coding genes 100,000 bp upstream and downstream of the target gene were considered as the cis‐target genes. The targets in trans were defined by calculating the expressed correlation with lncRNAs. Then, we performed GO and KEGG analysis of the DE lncRNA targets and mRNAs, respectively, using the in‐house scripts. The significance was expressed as FDR <0.05.

### 2.6 Construction of the co‐expression network

To gain a better understanding of interactions between the DEGs and DE lncRNAs, the Pearson correlation coefficient (COR) of mRNA‐lncRNA co‐expression network was calculated. Finally, the mRNA‐lncRNA co‐expression network was constructed using Cytoscape (version 3.7.2) with an absolute value of COR ≥0.7.

## 3 RESULTS AND DISCUSSION

### 3.1 RNA‐Seq analysis

The results of the RNA‐Seq reads mapping are shown in Table 1. To identify the potential function of lncRNAs in tail fat tissues, the nine cDNA libraries were sequenced using the Illumina Hiseq 4000 platform. A total of 139.82 G raw data were generated from the nine adipose tissues. After filtering out low‐quality reads, 131.61 G valid data were obtained, and the average valid ratio (reads) was 94%. In detail, the valid reads obtained were as follows: (1) 94,550,880; 98,994,810; and 99,167,036 per fat‐tail tissue sample from 6 M (A1, A2, and A3), (2) 96,734,688; 97,915,792; and 95,384,562 per fat‐tail tissue sample from 18 M (B1, B2, and B3), and (3) 98,530,172; 95,442,058; and 100,597,240 per fat‐tail tissue sample from 30 M (C1, C2, and C3), respectively. The average percentage of Q20 and Q30 base was more than 99% and 98%, respectively, and the percentage of the guanine–cytosine (GC) content of each sample on an average was 48%. Above all, we indicate that results of the RNA sequencing were highly reliable, and follow‐up analysis can be carried out.

**TABLE 1:** Summary of the reads mapped to the tail adipose tissue transcriptomes

Sample	Raw data		Valid data		Valid ratio (reads)	Q20%	Q30%	GC content%
	Read	Base	Read	Base				
A1	101,449,274	15.22G	94,550,880	14.18G	93.20	99.74	98.48	48
A2	103,736,482	15.56G	98,994,810	14.85G	95.43	99.81	98.63	48
A3	105,051,744	15.76G	99,167,036	14.88G	94.40	99.77	98.51	48
B1	102,382,092	15.36G	96,734,688	14.51G	94.48	99.76	98.47	48
B2	103,267,850	15.49G	97,915,792	14.69G	94.82	99.79	98.64	49
B3	102,376,260	15.36G	95,384,562	14.31G	93.17	99.70	98.36	47
C1	105,462,958	15.82G	98,530,172	14.78G	93.43	99.70	98.34	48
C2	101,700,360	15.26G	95,442,058	14.32G	93.85	99.71	98.35	49
C3	106,595,358	15.99G	100,597,240	15.09G	94.37	99.67	98.37	47

Note

A1, A2, and A3 are 6 months of age; B1, B2, and B3 are 18 months of age; C1, C2, and C3 are 30 months of age; valid ratio (reads) = (valid reads/raw reads).

### 3.2 Summary of lncRNA and mRNA expression

**FIGURE 1:**
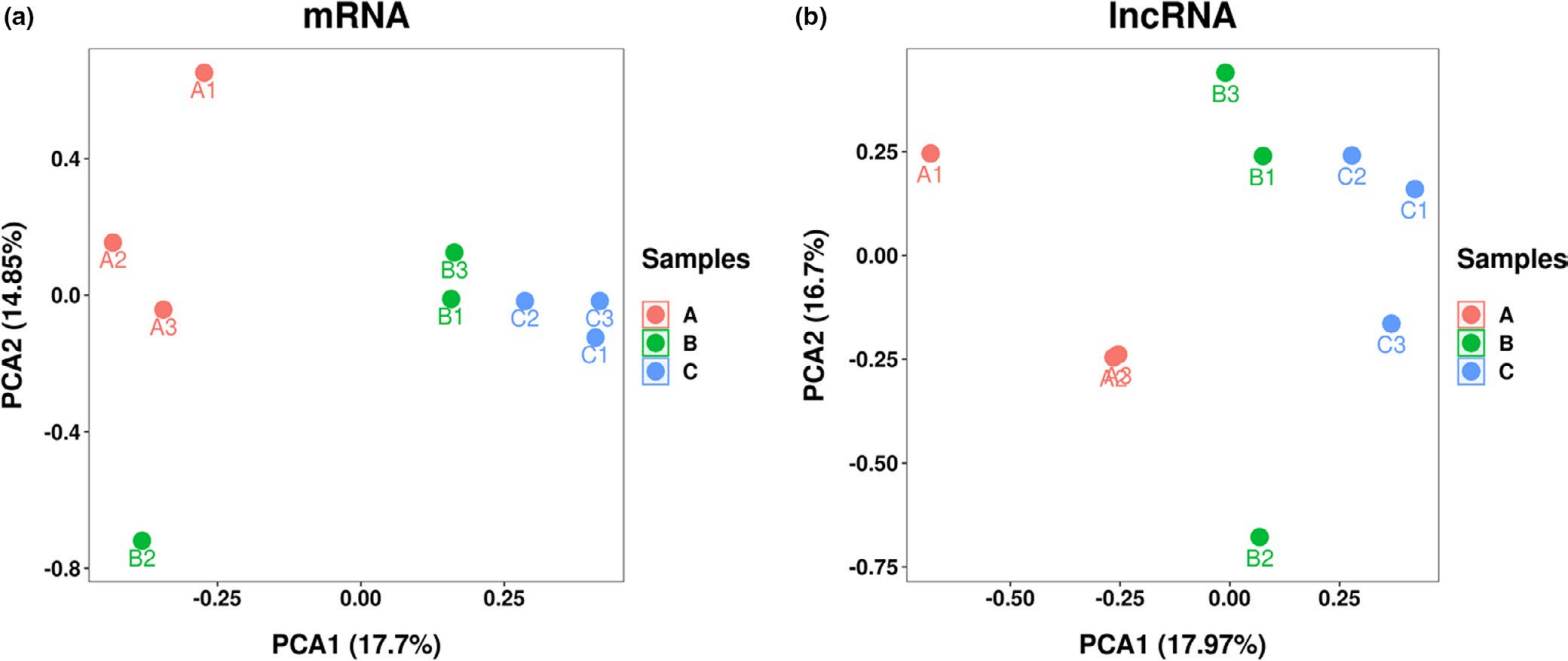
Principal component analysis for the mRNA (a) and lncRNA (b) in the sheep adipose tissue. 6 M: A1, A2, A3; 18 M: B1, B2, B3; 30 M: C1, C2, C3

**FIGURE 2:**
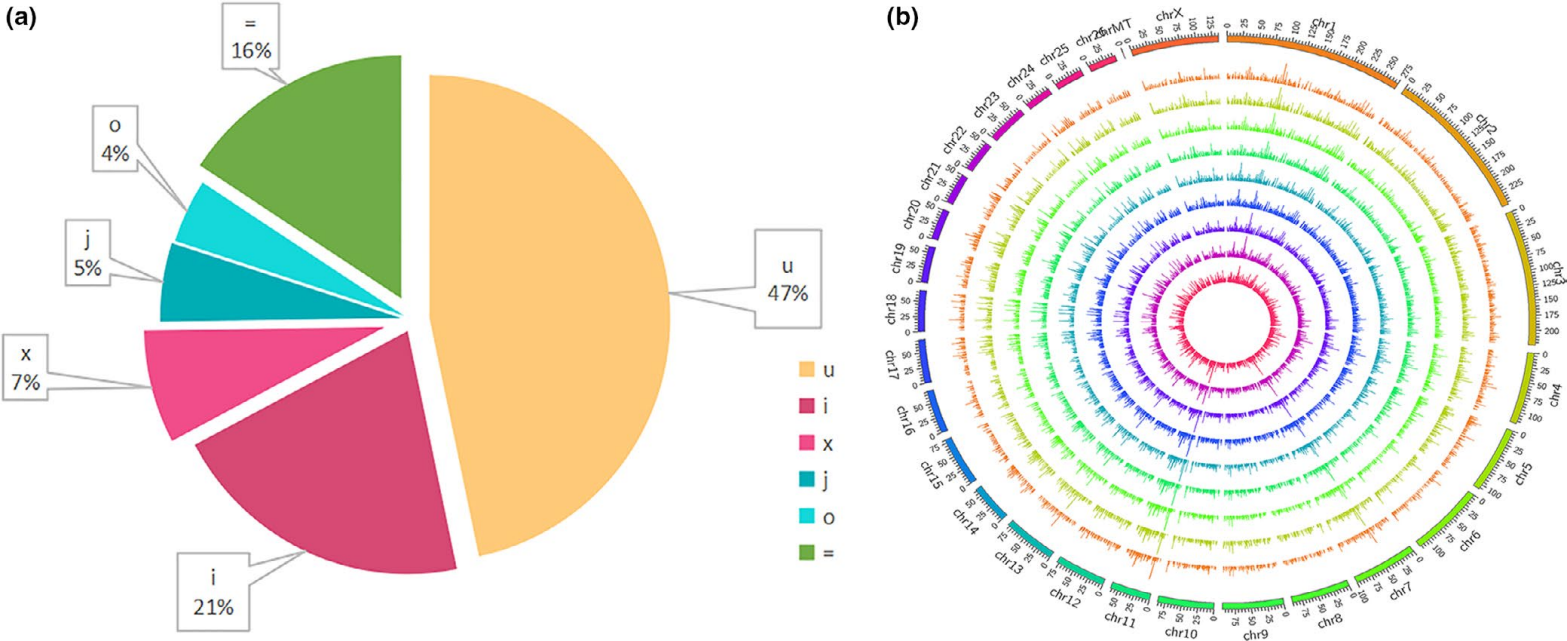
(a) Classification of lncRNA: (i) a transcript falling entirely within a reference intron (intronic); (j) potentially novel isoform or fragment at least one splice junction is shared with a reference transcript; (o) generic exonic overlap with a reference transcript; (u) unknown, intergenic transcript (intergenic); (x) exonic overlap with reference on the opposite strand (antisense); (=) complete match, considered as known lncRNAs. (b) The chromosome distribution of lncRNA. From outside to inside are samples 6 M (1–3), 18 M (1–3), and 30 M (1–3)

**FIGURE 3:**
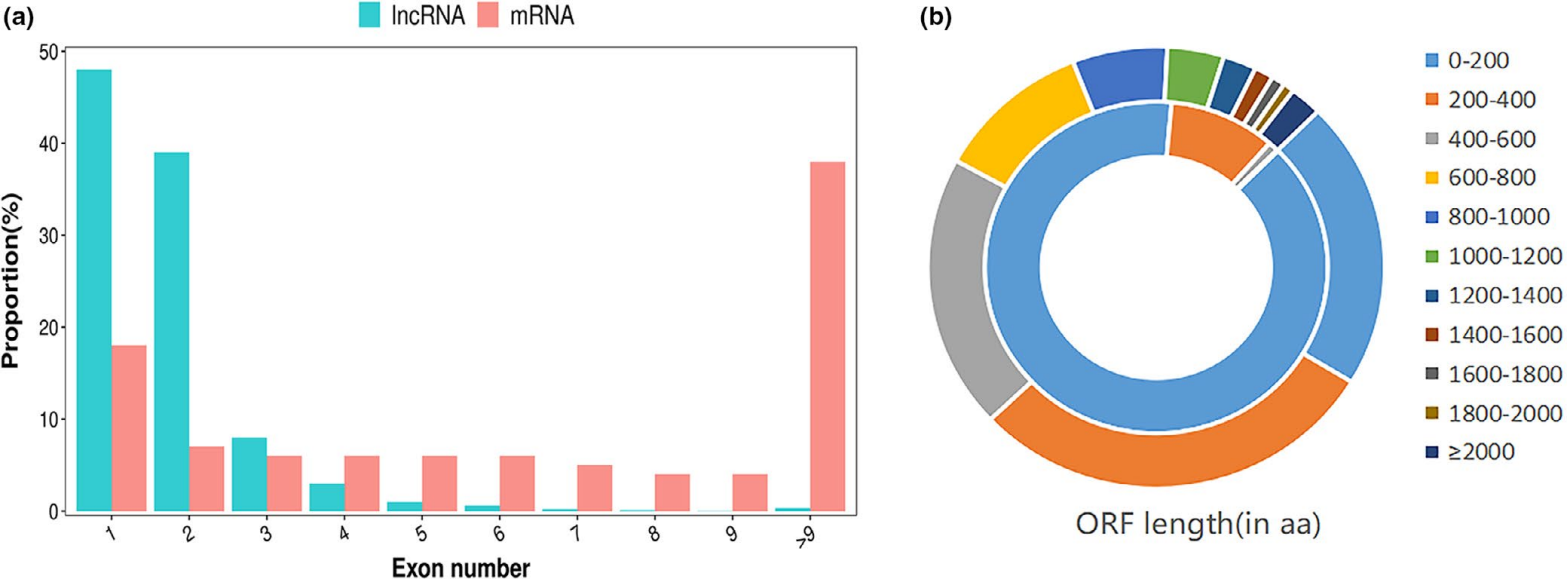
(a) Comparison of the number of exon in mRNAs and lncRNAs. (b) Comparison of the ORF length of mRNAs (outer) and lncRNAs (inner)

**FIGURE 4:**
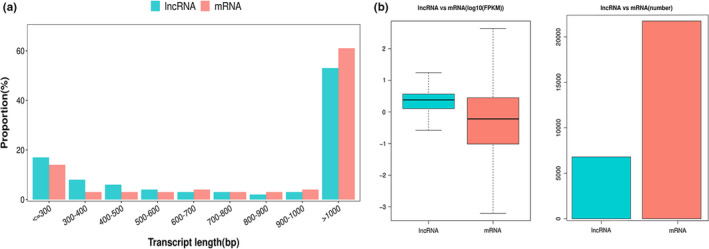
(a) Comparison of the transcript length of mRNAs and lncRNAs. (b) Comparison of the expression level and number of mRNAs and lncRNAs

To understand the expression profile of the lncRNAs in the tail fat tissue of SS, we identified the expression levels of the lncRNAs and compared them with the expression levels of mRNA. First, a total of 20,670 mRNAs and 6794 lncRNAs were identified. Here, 5722 lncRNAs were identified as novel, and the remaining 1702 lncRNAs were identified as known, which is more than the number of lncRNAs present in chicken (Muret et al., 2017) and cattle (Jiang et al., 2020) adipose tissue. The principal component analysis in mRNA (Figure 1a) and lncRNA (Figure 1b) showed that 18 M and 30 M were clustered together, while 6 M was separated from them, which suggests that 6 M has differences with 18 M and 30 M at the expression level. According to the classification rules, we classify novel lncRNAs as 1,395 (21%) class i, 354 (5%) class j, 288 (4%) class o, 3,174 (47%) class u, 511 (7%) class x, and 1,702 (16%) class = as known lncRNA (Figure 2a). The chromosome distribution of lncRNAs is shown in circos figure (Figure 2b). We found that most of the lncRNAs were mainly enhanced in chromosomes 1, 2, and 3. Then, lncRNAs and mRNAs were compared with exon number, open reading frames (ORF) length, transcript length, and expression levels. The mRNAs and lncRNAs had 9.7 and 1.8 exons on an average; 86% of lncRNAs contained 1–2 exons, and 38% of mRNAs contained more than 9 exons (Figure 3a). The size of the ORF of lncRNAs and mRNAs is mainly concentrated in the range of 0–200 and 0–600 amino acids, respectively (Figure 3b). The majority of lncRNAs and mRNAs were >1000 bp in size, and short‐range (≤300–600 bp) lncRNAs were more than mRNAs. The average length of lncRNAs and mRNAs was 3184 bp and 1903 bp, respectively. This significant difference might be due to the quantity gap of lncRNA and mRNA under similar distribution patterns (Figure 4a). The expression levels of lncRNA were higher than the expression levels of mRNAs (Figure 4b), which suggest that the lncRNAs may play an important role in the development of sheep tail fat tissue.

### 3.3 Different expression analysis

**FIGURE 5:**
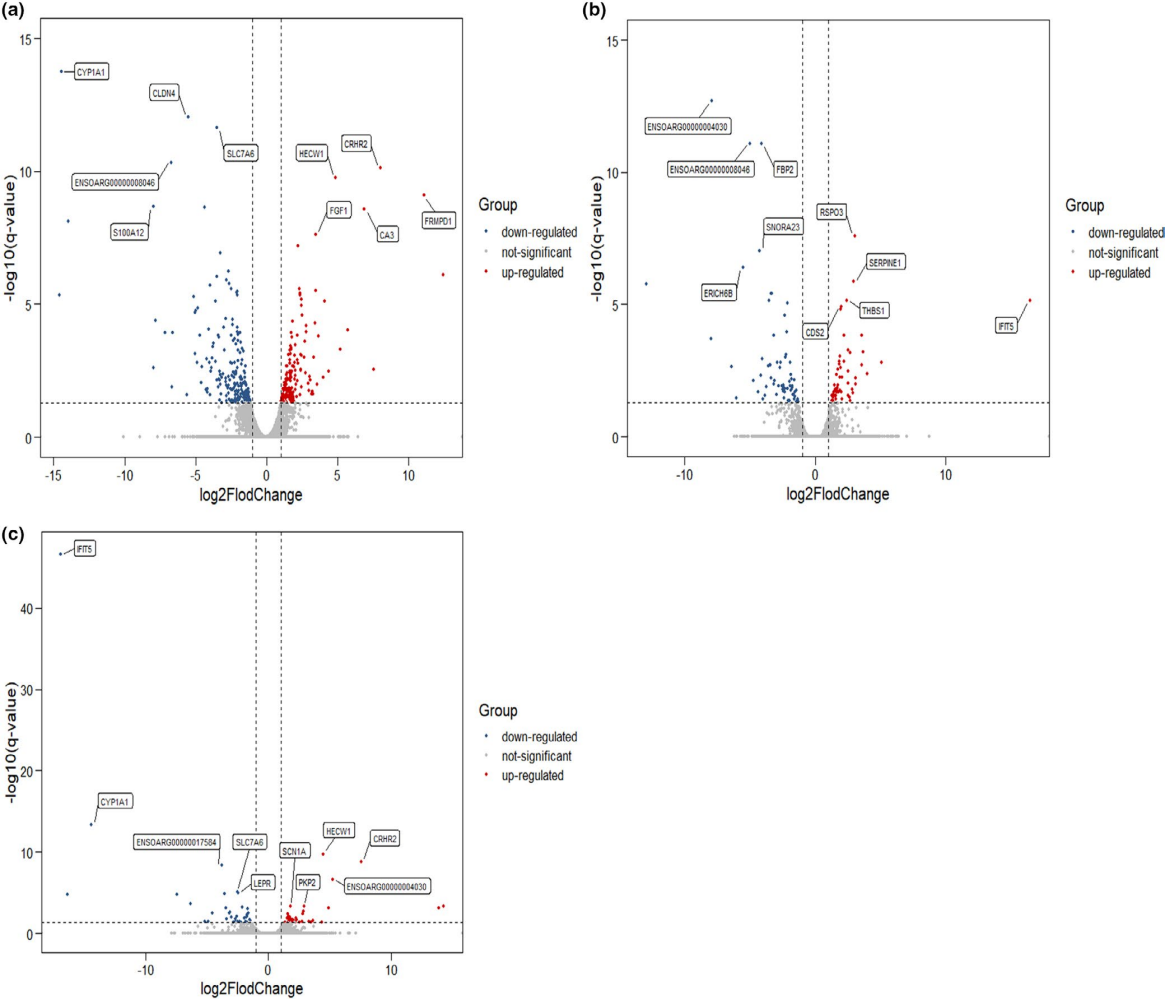
The volcano plot of DEGs. (a): 30 M versus 6 M; (b): 30 M versus 18 M; (c): 18 M versus 6 M. Annotated as the top five of the q‐value

**FIGURE 6:**
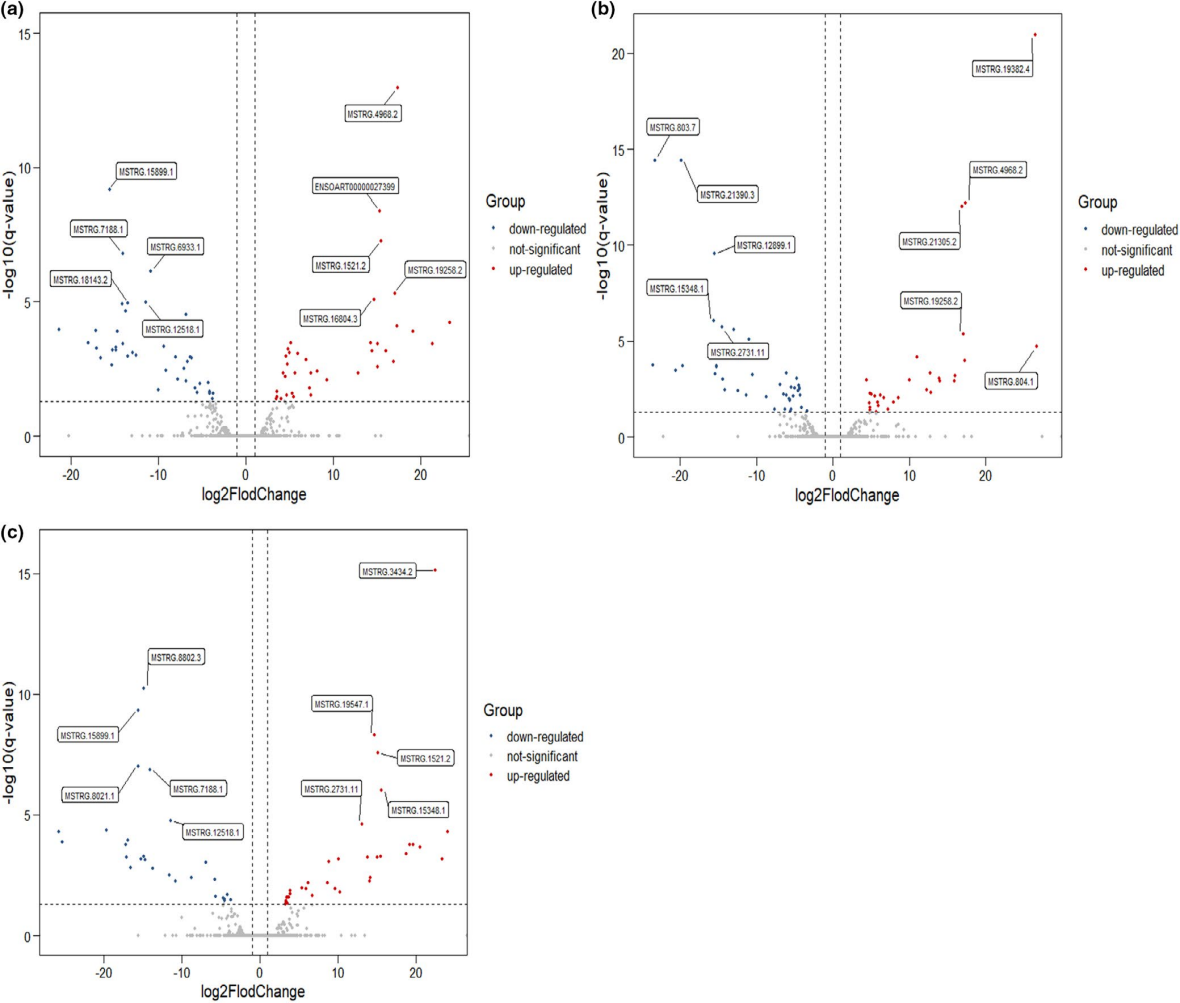
The volcano plot of DE lncRNAs. (a): 30 M versus 6 M; (b): 30 M versus 18 M; (c): 18 M versus 6 M. Annotated as the top five of the q‐value

We compared the expression profiles between any two stages (30 M versus 18 M, 18 M versus 6 M, and 30 M versus 6 M) using |log_2_(Fold Change) | ≥1 and FDR <0.05 to identify DEGs and DE lncRNAs. In the comparison between 30 M versus 6 M, we found 377 DEGs (167 upregulated and 210 downregulated genes). In the 30 M versus 18 M group, 125 DEGs (56 upregulated and 69 downregulated genes) were obtained. In the comparison of 18 M versus 6 M, 75 DEGs (38 upregulated and 37 downregulated) were found (Figure 5). Furthermore, 4 DEGs were commonly expressed in the comparison groups of 30 M versus 18 M and 18 M versus 6 M, including *IFIT5*, *THBS1*, *ENSOARG00000004030*, and *ENSOARG00000018868*. Sixty‐eight DEGs were commonly expressed in 30 M versus 6 M and 30 M versus 18 M, and 35 DEGs were commonly expressed between 30 M versus 6 M and 18 M versus 6 M. On the other hand, 151 DE lncRNAs were identified in 30 M versus 6 M, 30 M versus 18 M, and 18 M versus 6 M. Among them, 78 DE lncRNAs including 38 upregulated (36 novel, 2 known) and 40 downregulated (39 novel, 1 known), 71 DE lncRNAs including 30 upregulated (30 novel, 0 known) and 41 downregulated (38 novel, 3 known), 61 DE lncRNAs including 34 upregulated (33 novel, 1 known) and 27 downregulated (25 novel, 2 known), respectively (Figure 6). Fifteen DE lncRNAs were commonly expressed in the comparison groups of 30 M versus 18 M and 18 M versus 6 M, 25 DE lncRNAs were commonly expressed in 30 M versus 6 M and 18 M versus 6 M, and 19 DE lncRNAs were commonly expressed in 30 M versus 6 M and 30 M versus 18 M.

### 3.4 Functional analysis of DEGs

**FIGURE 7:**
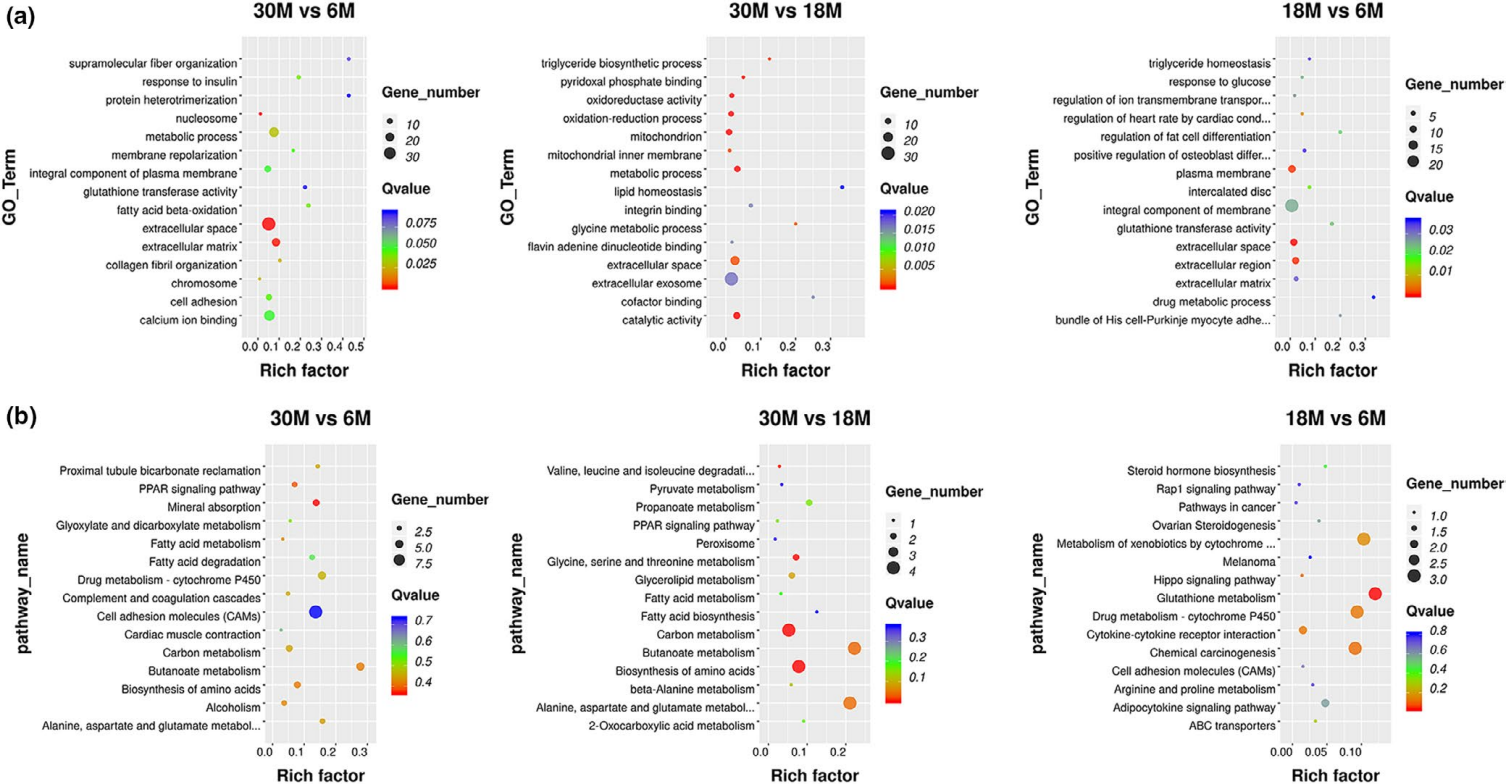
The top 15 GO and KEGG analysis of DEGs (a) GO analysis of three comparison groups; (b) KEGG analysis of three comparison groups

The top 15 GO terms and KEGG Pathway analysis performed in DEGs of the fat‐tail tissue at three different growth stages are shown in the scatterplot (Figure 7). GO terms were determined by three functions, including cellular component, biological process, and molecular function. We found that more than half of the GO terms were enriched in the biological process in the comparison groups, and the cellular component obtained the most number of genes in the three comparison groups. In the three stages, the DEGs were significantly enriched in 80 GO terms, and several fat‐related functions were obtained, including fatty acid beta‐oxidation, triglyceride biosynthetic process, triglyceride homeostasis, lipid homeostasis, lipid biosynthetic process, and regulation of fat cell differentiation, suggesting that these functions might contribute to the development of the sheep tail fat. We found five highly expressed DEGs, namely *EHHADH*, *LPIN1*, *ACACA*, *THRSP*, and *GPAT4*, which were related to these functions. The previous study shows that *LPIN1* deficiency will lead to a significant decrease in adipose tissue and abnormal expression of adipogenic genes. Conversely, increased expression of *LPIN1* in skeletal muscle or adipose tissue will promote obesity in mice (He et al., 2009). *EHHADH* is associated with the expression of genes involved in the tricarboxylic acid cycle, mitochondrial and peroxisome fatty acid oxidation, and is indispensable for the production of medium‐chain dicarboxylic acids in mice during fasting (Houten et al., 2012). *ACACA* is considered to be a key regulator of fat production and a limiting factor in the synthesis of long‐chain fatty acids. Acetyl‐CoA can be converted to malonyl‐CoA (Pena et al., 2019), which may play a key role in energy metabolism and homeostasis in sheep tail fat cells. *THRSP* is involved in the process of adipogenesis in rodents, and it may be a potential marker gene for bovine intramuscular fat. Studies have shown that *THRSP* is mainly expressed in adipocyte nuclei, intramuscular adipocytes, and related cells and expressed in mature adipocytes rather than in the early stages of adipogenesis (Schering et al., 2017). In our study, the THRSP gene with a higher expression level (FPKM) of 324 and 301 at 6 M and 18 M, averagely, and significantly decreased at 30 M with an average expression level (FPKM) of 77. We can speculate that in the early fat tissue of sheep's tail, fat hypertrophy is mainly manifested by the increase in the number of fat cells, and as the age increases, fat hypertrophy is reflected by the increase in the volume of fat cells. In addition, there were also some highly expressed genes related to fat metabolism, such as *GPAT4*, *ACSM1*, *ACSM3*, *ACAT1*, *TKT*, and *ECHS1*. *GPAT4* was reported to be responsible for maintaining triacylglycerol stores (Cooper et al., 2015), and *ACSM1*, *ACSM3*, and *ACAT1* were related to fat deposition and fatty acid metabolism (Berton et al., 2016; Guo et al., 2014; Huang, Guo, et al., 2017). *ECHS1* was shown to be associated with the fatty acid beta‐oxidation (Peng et al., 2016). Studies have shown that *TKT* expression affects fatty acid oxidation and mitochondrial function (Tian et al., 2020). On the other hand, a total of 8 KEGG pathways were significantly enriched in three different stages. They were mainly focused on metabolism processes, including carbon metabolism, mineral absorption, glutathione metabolism, butanoate metabolism, and some related amino acid metabolism. Based on the KEGG pathway analysis, those highly expressed DEGs were related to butanoate metabolism, fatty acid metabolism, glycerolipid metabolism, and PPAR signaling pathway, which may contribute to the fat deposition in sheep tail fat.

### 3.5 Hierarchical clustering analysis of fat‐related DEGs

**FIGURE 8:**
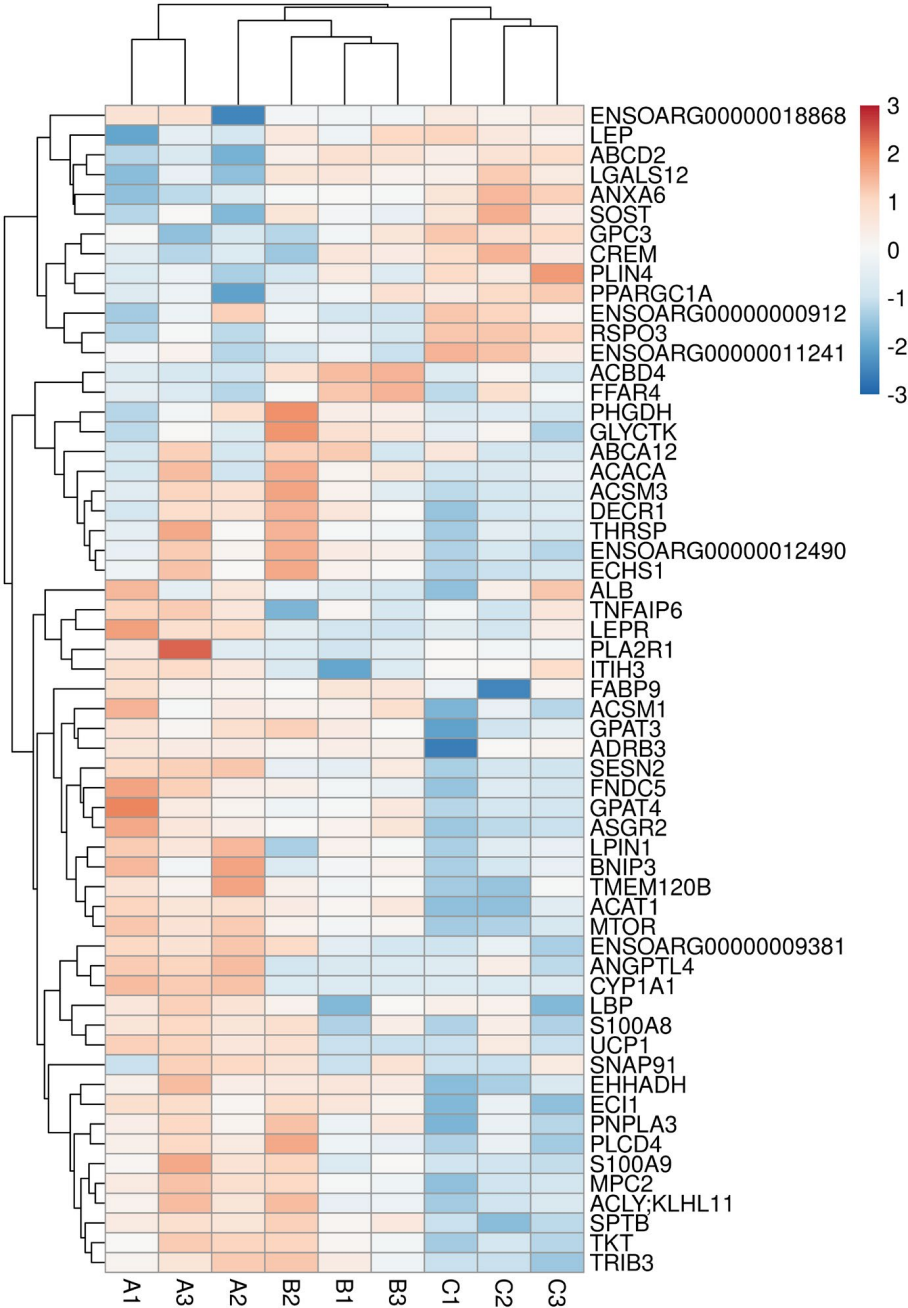
Heatmap of fat development related DEGs

Based on the above analysis and further screening, we obtained several DEGs that may be related to fat‐tail development. We performed a hierarchical clustering analysis to show the expression patterns of these DEGs (Figure 8). It is not difficult to find that most of the genes are active in the early months of age, especially during the 6 M, and the expression of DEGs decreased gradually with the increase in age. Therefore, we indicate that the vitality of fat development weakens with the increase of age, that is to say, the development of tail fat will be more active at the age of 6 M, but will gradually decrease at the age of 18 M and 30 M. There is a significant difference between 6 M and 30 M of age. Further, the expression pattern at 18 M, as the middle month, plays the role of transitioning from high metabolic activity to low metabolic activity. However, our findings are only possible in theory, and the mechanism needs to be further identified.

### 3.6 Target gene prediction and functional analysis

**FIGURE 9:**
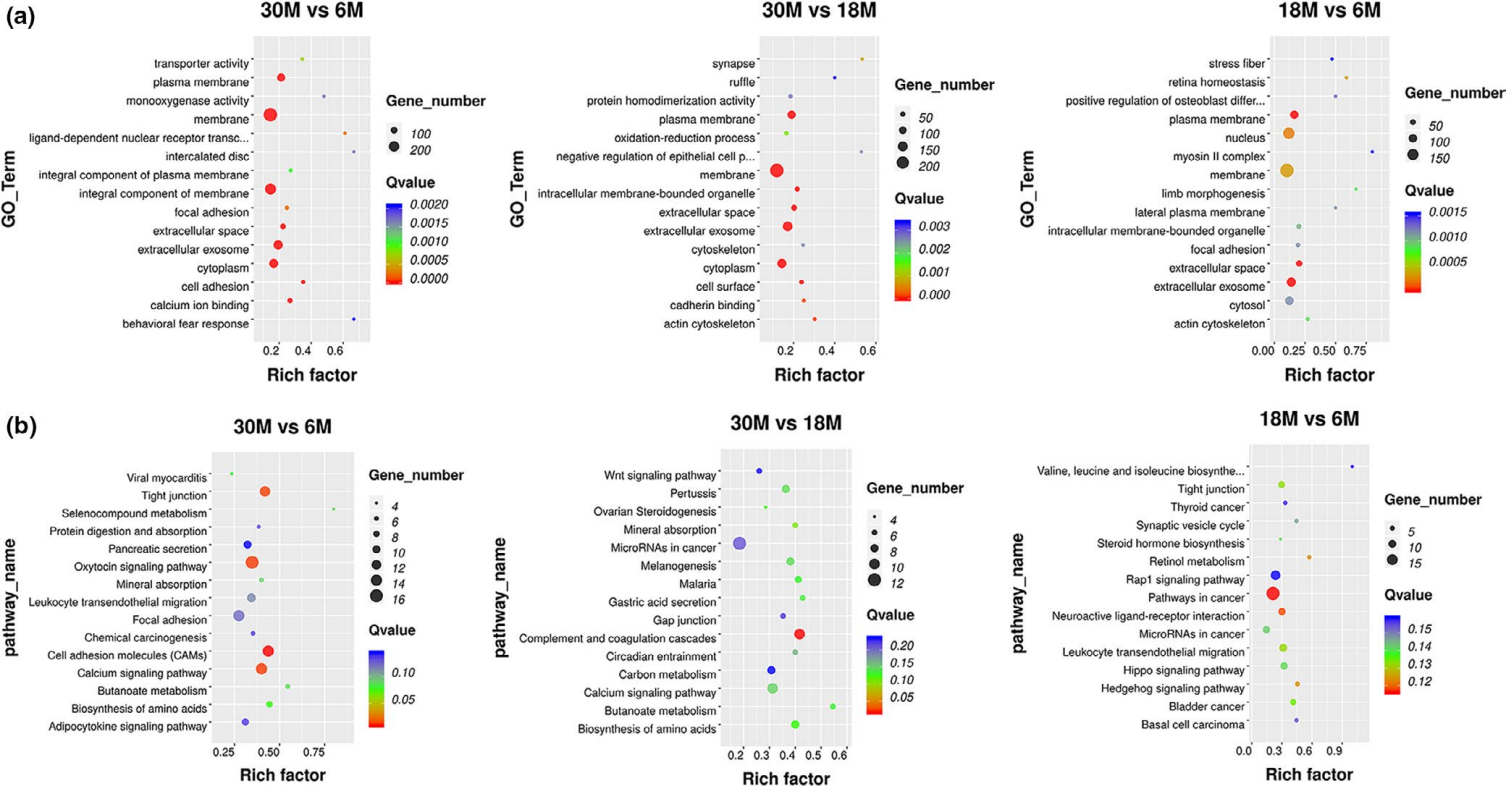
The top 15 GO and KEGG analysis of DE lncRNAs. (a) GO analysis of three comparison groups; (b) KEGG analysis of three comparison groups

In order to explore how lncRNA participates in regulation, we predicted DE lncRNAs based on cis‐ and transregulation in three different stages of the fat‐tail development. In our study, 148 DE lncRNAs (68 upregulated and 80 downregulated) were obtained and the target genes prediction analysis was performed in these DE lncRNAs. A total of 186, 113, and 150 GO terms were significantly enriched in target genes of 30 M versus 6 M, 30 M versus 18 M, and 18 M versus 6 M (FDR < 0.05), respectively. The top 15 GO terms and KEGG pathway of target genes of DE lncRNAs in the three comparison groups are shown in the scatterplot (Figure 9). There were 5 common GO terms enriched in the three comparison groups, namely plasma membrane, extracellular exosome, membrane, extracellular, and cytoplasm. The target genes of DE lncRNAs in 30 M versus 6 M were significantly enriched in 4 KEGG pathways, including calcium signaling pathway, cell adhesion molecules, oxytocin signaling pathway, and tight junction. Among these DE lncRNA, only one cis‐regulated target gene was obtained: *MSTRG.13384.1* targets *CLDN4*. We found that most of the lncRNAs were targets to more than 20 mRNAs as their transregulators, *MSTRG.20969.1* targets to 53 mRNAs, as the largest number in 30 M versus 6 M. The most commonly enriched top 5 target genes were *SLC7A6* (38 DE lncRNA), *CDS2* (32 DE lncRNA), *CA3* (31 DE lncRNA), *SLC6A2* (31 DE lncRNA), and *PRTG* (30 DE lncRNA). These target genes were mainly enriched in cellular components, such as membrane and integral component of membrane. Previous studies have indicated that with obesity, the concentration and activity of *CA3* in rat adipose tissue decreased (Wang et al., 2006). The complement and coagulation cascades are the only KEGG pathway that is significantly enriched in 30 M versus 18 M. In this comparison group, *MSTRG.12899.1* and *ENSOART00000028120* were connected to 38 mRNAs as the largest number in 30 M versus 18 M, respectively. The most commonly enriched top 5 target genes were *SNORA23* (29 DE lncRNA), *ERICH6B* (27 DE lncRNA), *ENSOARG00000018868* (22 DE lncRNA), *FBP2* (22 DE lncRNA), and *ENSOARG00000014791* (16 DE lncRNA), among which *ENSOARG00000018868* is related to lipid binding. In 18 M versus 6 M, *MSTRG.14210.1* targets to 48 mRNAs as the largest number. The most commonly enriched top 5 targets genes were *HECW1* (23 DE lncRNA), *CRHR2* (19 DE lncRNA), *FRK* (19 DE lncRNA), *IFIT5* (19 DE lncRNA), and *PTPRZ1* (19 DE lncRNA). Based on the GO analysis, these target genes were mainly enriched in molecular function, including ATP binding and Hippo signaling pathway, and steroid hormone biosynthesis was obtained in KEGG analysis.

Further, we obtained several fat‐related DE target genes in these three comparison groups. Among these targets DEGs, some of them were regarded as common DE target genes in two comparison groups, including *TRIB3, ACSM1, ACSM3, TKT, SPTB,* and *ASGR* in 30 M versus 6 M and 30 M versus 18 M, and *CYP1A1* and *LBP* in 30 M versus 6 M and 18 M versus 6 M. Based on the GO analysis, these DEGs were mainly enriched in fatty acid ligase activity, fatty acid biosynthetic process, glyceraldehyde‐3‐phosphate biosynthetic process, negative regulation of fat cell differentiation, and some lipid related functions, such as lipid binding and lipid homeostasis. It has been reported that *TRIB3* was might inhibit subcutaneous fat deposition in Large White pig, and lncRNA *XLOC_064871* transregulates *TRIB3*, so *XLOC_064871* might play an important role in adipocyte differentiation and fatty acid metabolism in pig (Huang et al., 2017). *CYP1A1* is only expressed at 6 M in our study, and it was reported to be expressed in brown adipose tissue (Galván et al., 2005). The study showed that using a specific anti‐*LBP* antibody to inhibit *LBP* activity can improve the adipogenic status of fully differentiated adipocytes, which makes *LBP* is a novel adipokine that might display an essential role in inflammation and obesity‐associated adipose tissue dysfunction (Moreno‐Navarrete et al., 2013). In addition, we found that the same target gene was affected by different amounts and types of DE lncRNAs at different ages. For example, in 30 M versus 6 M and 30 M versus 18 M, 13 and 11 DE lncRNAs were connected to *ACSM1*, and there were four common lncRNA targets to *ACSM1* between two comparison groups; 13 and 15 lncRNAs were connected to *ACSM3*, and 22 and 19 lncRNAs were connected to *TKT* between two comparison groups; however, *MSTRG.3410.1* is the only one lncRNA that acts as a target to *ACSM3* and *TKT*, which suggest that *MSTRG.3410.1* may be related to the fat deposition. It could indicate that different lncRNAs with different regulation patterns may impact the target gene expression pattern and play its role in different growth stages of sheep tail fat.

### 3.7 Co‐expression network construction

**FIGURE 10:**
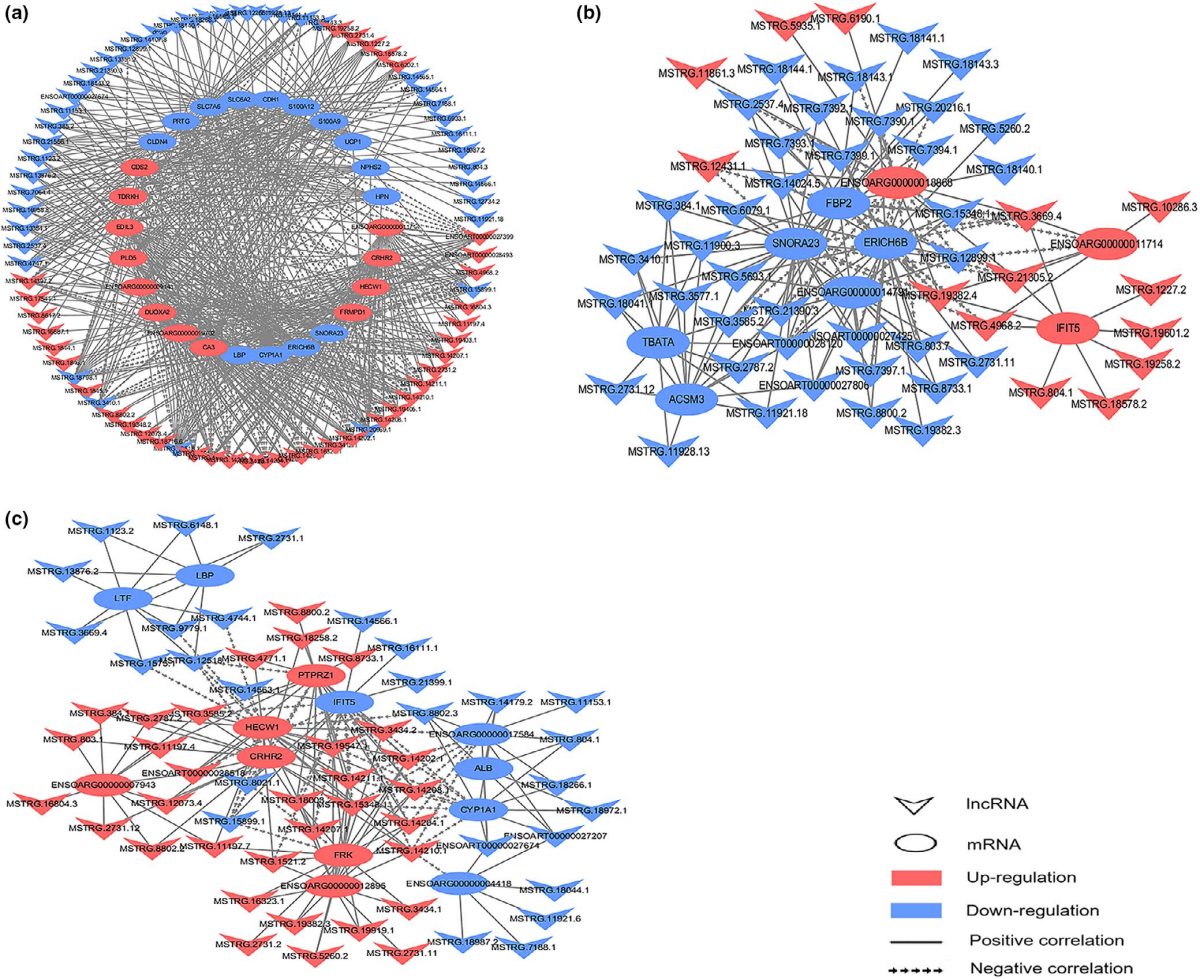
The co‐expression network of 30 M versus 6 M (a), 30 M versus 18 M (b), and 18 M versus 6 M (c)

We constructed three co‐expression networks based on DEGs and DE lncRNA in sheep fat tail using Cytoscape (version 3.7.2) (Figure 10). A total of 538, 158, and 184 pairs of co‐expression pairs were obtained in 30 M versus 6 M, 30 M versus 18 M, and 18 M versus 6 M, respectively. In the comparison of 30 M versus 6 M, 78 DE lncRNAs connected to the 26 mRNAs, and 538 pairs of co‐expression pairs were obtained (403 positively and 135 negatively correlated). There were 20 lncRNAs connected to more than 10 mRNAs. *MSTRG.20969.1* and *MSTRG.12518.1* were connected to mRNA 21 and 20, respectively. In the 30 M versus 18 M, 52 DE lncRNAs connected to the 9 mRNAs, and 149 pairs (118 positively correlated and 31 negatively correlated) were obtained. *ENSOART00000028120* and *MSTRG.19382.4* were co‐expressed with 7 mRNAs. In 18 M versus 6 M, 60 DE lncRNAs connected to the 13 mRNAs, and 184 pairs (139 positively correlated and 45 negatively correlated) of co‐expression pairs were obtained. *MSTRG.15348.1, MSTRG.14210.1*, and *MSTRG.14211.1* were co‐expressed with 8 mRNAs. In these two comparison groups, there were only 7 (30 M versus 18 M), and 8 (18 M versus 6 M) mRNAs connected to single lncRNA, at most. This indicate that these co‐expression pairs might play a crucial role, and lncRNA may regulate the development of sheep tail fat mainly through positive correlation with multiple mRNAs.

## 4 CONCLUSIONS

In our study, we used transcriptome analysis to explore the underlying molecular mechanism of different growth stages of SS tail fat. We identified 377 DEGs and 78 DE lncRNAs between the 30 M versus 6 M, 125 DEGs and 71 DE lncRNAs were found between the 30 M versus 18 M, and 75 DEGs and 61 DE lncRNAs were found between the 18 M versus 6 M (FDR < 0.05 and |Fold Change| ≥2), respectively. According to the GO and KEGG analysis of DEGs, we conclude that the fat deposition in the sheep tail may be active in the early stages of growth and gradually decrease with the increase of age, and 18 M may be a transitional period in this process. On the other hand, lncRNA participates in the regulation of the growth and development of tail fat by targeting the mRNA. These findings could provide a better understanding of the regulatory mechanism of sheep tail fat development and provide basic theoretical data for further research.

## CONFLICT OF INTEREST

None.

## AUTHOR CONTRIBUTION

**Xige He:** Conceptualization (equal); Data curation (equal); Formal analysis (equal); Investigation (equal); Methodology (equal); Software (equal); Validation (equal); Visualization (lead); Writing‐original draft (lead); Writing‐review & editing (equal). **Rihan Wu:** Formal analysis (equal); Methodology (equal); Software (equal). **Yueying Yun:** Formal analysis (equal); Software (equal); Validation (equal). **Xia Qin:** Formal analysis (equal); Methodology (equal); Software (equal). **Lu Chen:** Data curation (equal); Formal analysis (equal); Software (equal). **Yunfei Han:** Data curation (equal); Formal analysis (equal); Software (equal). **Jindi Wu:** Formal analysis (equal); Methodology (equal); Software (equal). **Lina Sha:** Conceptualization (equal); Investigation (equal); Methodology (equal). **Gerelt Borjigin:** Conceptualization (equal); Data curation (equal); Formal analysis (equal); Funding acquisition (lead); Investigation (equal); Methodology (equal); Project administration (lead); Resources (lead); Supervision (lead); Validation (equal); Writing‐review & editing (equal).

## ETHICAL APPROVAL

All experimental procedures were approved by the Animal Ethics Committee of the Inner Mongolia Agricultural University's Animal Experimentation Area and followed the Chinese Animal Protection Law.

## Data Availability

The datasets used and/or analysed during the current study are available from the corresponding author on reasonable request.
